# Molecular Characterization of New Delhi Metallo-ß-Lactamases-Producing Bacteria in the Gastrointestinal Tract of Intensive Care Unit Patients

**DOI:** 10.7759/cureus.12257

**Published:** 2020-12-24

**Authors:** Dipasri Konar, Naz Perween, Shyam Kishor Kumar, Prabhav Aggarwal, Beena Uppal

**Affiliations:** 1 Microbiology, Jan Swasthya Sahyog, Chhatishgarh, IND; 2 Microbiology, Superspeciality Paediatric Hospital & Postgraduate Teaching Institute, Noida, IND; 3 Microbiology, SRL Diagnostics, Deoghar, IND; 4 Microbiology, Maulana Azad Medical College, New Delhi, IND

**Keywords:** gastrointestinal carriage, carbapenem, ndm, icu

## Abstract

Background: The emergence of carbapenem-resistance in the gut flora of patients in the intensive care unit (ICU) poses a significant risk for infection with these types of pathogens.

Materials and methods: New Delhi metallo-ß-lactamase 1 (NDM-1) in the gut flora of ICU patients was detected in cultures of a single rectal swab from each patient admitted to the ICU for a minimum period of 48 hrs. Samples were processed in the microbiology laboratory using blood agar and MacConkey agar. Identification of pathogens, carbapenem resistance, and metallo-ß-lactamase production was made using standard laboratory procedures. Bacterial isolates were also used for the determination of the NDM-1 gene by molecular methods.

Results: One hundred twenty-two patients with different clinical presentations were recruited in the study. Two hundred nine bacteria were isolated, with *Escherichia coli* being the most common isolate. A total of 54/122 (44.3%) patients harbored carbapenem-resistant organisms (CRO), 36/122 (29.5%) carried metallo-β-lactamase-producing organisms (MBLO), and 30/122 (24.6%) carried bacteria with the NDM-1 gene. Patients who harbored CRO and MBLO had longer mean duration of stay in the ICU and hospital than those not harboring CRO and MBLO. All the metallo-β-lactamases were simultaneously resistant to other groups of antibiotics also. Use of invasive devices, three or more classes of antibiotics, hospitalization during the previous six months, comorbidities, and hospital stay for ≥48 hours before ICU admission had a significant association with colonization with CRO.

Conclusion: Patients admitted in ICU or with serious diseases should be screened for gastrointestinal carriage of carbapenem-resistant organisms. Irrational use of antibiotics must be stopped to prevent the emergence and spread of such organisms.

## Introduction

Antimicrobial-resistant pathogens, confronted in the highest numbers in intensive care units (ICU) within hospitals, are a pervasive problem and affect the clinical outcome of the patients admitted. India carries a big part in the global burden of antibiotic resistance [1]. During the last three decades, the efficacy of ß-lactam drugs has been much constrained due to the emergence of extended-spectrum ß-lactamases (ESBLs)-producing bacteria. The appearance of carbapenemases-producing bacteria has further compounded this problem [2]. There are two principal mechanisms for carbapenem resistance- that due to the production of carbapenem hydrolyzing enzymes (serine carbapenemases and metallo-ß-lactamases) and that due to the combination of membrane impermeability with the production of ESBLs, pAmpC or ampC overexpression. The former is more important because these enzyme-producing genes are encoded by mobile genetic elements. These are carried on a plasmid and associated with resistance genes for other antibiotics like aminoglycosides, tetracyclines, folate inhibitors, and fluoroquinolones [2,3]. Debilitated patients, old age, patients exposed to invasive procedures, or indwelling devices are at constant risk of infections with these resistant organisms. These conditions are often found in ICU settings. The prevalence of carbapenem resistance is reported from 5.9% to 59% in various studies in India [4,5]. Carbapenemases belong to all four classes of ß-lactamases, A, B, C, and D. Class B metallo-ß-lactamases include NDM, VIM, and IMP family enzymes and hydrolyzing most of the ß-lactams including carbapenems. New Delhi metallo-ß-lactamase 1 (NDM-1) is a crucial enzyme with widespread distribution in the Indian subcontinent. It was first reported in 2007, in a patient of Indian origin in Sweden and is now reported in more than 15 countries [2]. Theoretically, colonization with a pathogen is a prerequisite for subsequent invasive disease. It has been hypothesized that colonization surveillance in such patients can provide early insight into the microbial etiology of subsequent infection, allowing thus the provision of adequate empiric treatment in a timely fashion [6].

This study aimed to find the prevalence of gastrointestinal carriage of carbapenem-resistant organisms (CRO), metallo-β-lactamase producing organisms (MBLO), and NDM-1 in ICU patients with various risk factors associated with this condition.

## Materials and methods

A prospective cross-sectional study was carried out in the Department of Microbiology in conjunction with the Department of Anaesthesia and Critical Care, Maulana Azad Medical College and associated Lok Nayak Hospital, New Delhi, over two years. The study was ethically approved. Patients >18 years of age admitted in ICU for >48 hours included in the study while patients <18 years and ICU staying hours <48 hours were excluded from the study. After taking informed consent, a detailed history of the patient profile and various risk factors associated with the development of multidrug resistance in an ICU was filled up in a preformed proforma. Rectal swab (laboratory prepared cotton swab- a wooden stick of six to eight inches wrapped with cotton at the tip) was used for taking samples. Inoculation of the sample was done on blood agar & MacConkey agar (Himedia, Mumbai, India) at the bedside to minimize the loss of organisms during transport. Inoculated plates were further processed in the microbiology laboratory and incubated at 37℃ for 18-24 hours. Isolated bacteria were identified on colony morphology, biochemical characteristics and confirmed by slide agglutination where required as per standard guidelines. 

All isolates were tested for resistance to antibiotics (penicillins, aminoglycosides, fluoroquinolones, carbapenems, cephalosporins, tetracyclines.) as per Clinical & Laboratory Standards Institute (CLSI) guidelines [7].

Screening of isolates for carbapenem resistance

The isolates were screened for carbapenemase production using the initial screening test as recommended by the CLSI guidelines, 2011. Isolates were inoculated on Muller-Hinton agar and tested by disc-diffusion using a 10μg disc of meropenem. A zone size of <21 mm was taken as carbapenem-resistant.

Phenotypic confirmatory test for production of metallo-β-lactamase (MBL)

**Figure 1:**
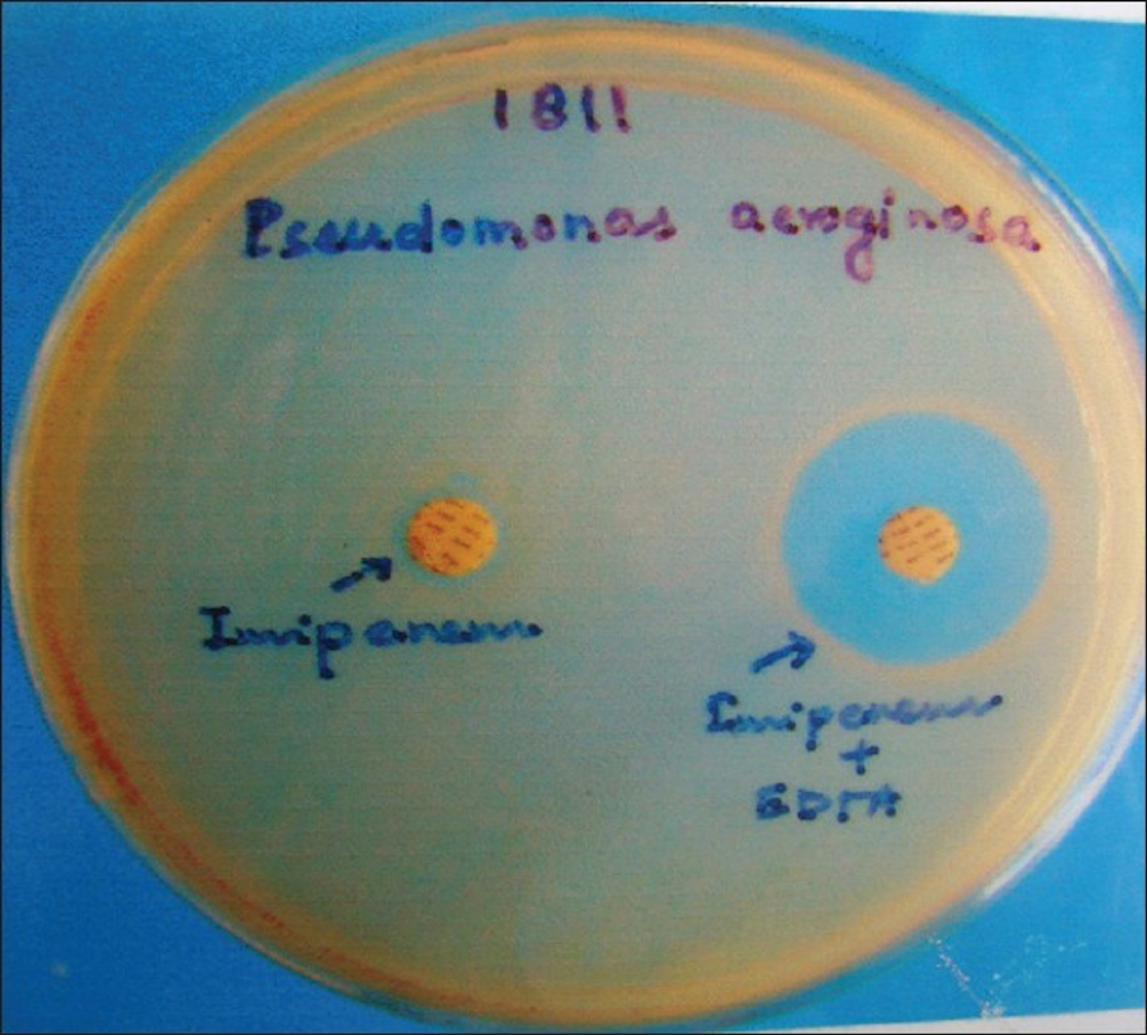
Showing double disc diffusion test

All isolates with positive screening tests were evaluated for MBL production using a combination of imipenem and imipenem-EDTA by Double Disk Diffusion Technique. Two imipenem discs (10μg), one containing 10μg of 0.1M anhydrous EDTA (292μg), were placed 25mm apart on Mueller Hinton agar plates. A strain producing a diameter of >4mm around the disc with IMP-EDTA and not around the disc with IMP alone was considered positive for MBL production (Figure 1).

Table 1 shows detection of the NDM-1 gene using conventional polymerase chain reaction (PCR) technique.

**Table 1:** Primer sequence/PCR conditions for the blaNDM-1 resistance genotyping

Gene	Primer Sequences (5’-3’)	Annealing Temperature	No. of cycles	Amplicon size (bp)	Reference no
NDM-1	F:GGGCAGTCGCTTCCAACGGT R:GTAGTGCTCAGTGTCGGCAT	60	30	475	[8]

DNA extraction

Deoxyribonucleic Acid (DNA) extraction was carried out using the MagnaPure Compact automated nucleic acid extraction system (Roche Diagnostics, Rotkreuz, Switzerland) as per manufacturer protocol to isolate bacterial whole-cell nucleic acid. The extracted DNA was stored at -20°C and used for various molecular studies.

PCR reactions for NDM-1

Briefly, PCR reactions were performed in a final volume of 25 μl of the amplification mixture containing 1.25 U of Taq DNA polymerase, 1X Taq buffer, 0.2 mM each of dNTPs, 0.2 μM of each primer, and two μl of DNA template. The PCR was carried out with a Veriti Thermal using the following conditions: 94°C for10 min; 94°C for 30 sec, 60°C for 40 sec, and 72°C for 1 min for 30 cycles, with a final extension at 72°C for 7 min. PCR products were visualized on a 1.8% agarose gel stained with ethidium bromide.

Sequencing

Thirty blaNDM-1 positive* E. coli* strain PCR-amplified products were sequenced by Applied Biosystems 3500xL Genetic Analyzer (Waltham, Massachusetts, USA). Nucleotide sequence similarity searches were performed using the National Centre for Biotechnology Information (NCBI) (https://blast.ncbi.nlm.nih.gov/Blast.cgi). BLAST, CLUSTALX, and MEGA 10.0.5 software was used for sequence alignment of the amplicon sequence obtained with already submitted sequences of blaNDM-1 in GenBank. The sequences obtained by sequencing have been submitted to GenBank under accession number 2345003.

Statistical analysis

The data was scrutinized, coded, and fed into Microsoft Excel sheets 2010 and analyzed in Statistical Package for Social Sciences (SPSS) Statistics version 17 (IBM Corp., Armonk, NY, USA) and EPI INFO 2005 software of the World Health Organization. Data are expressed in terms of percentages. Fisher’s exact test observed differences between the proportions. 

## Results

One hundred twenty-two patients of different clinical presentations were recruited for the study after taking informed consent and fulfilling the inclusion criteria. There were 54 females and 68 males of different age groups (Table 2). Sixty-four patients were admitted directly to the ICU. Fifty-eight were admitted to the ICU after a period of hospitalization varying from one to 27 days. The mean age was 36.9 years. The mean duration of ICU stay was 8.9 days, and the mean duration of stay in the hospital was 11.6 days. 

**Table 2:** Showing age/sex distribution of patients CRO = carbapenem-resistant organisms

Age in years	No. of male patients	No. of female patients	No. of CRO in males	No. of CRO in females
18-20	14	16	6	8
21-30	14	12	6	2
31-40	11	6	6	3
41-50	7	9	4	6
51-60	11	6	3	3
>60	11	5	6	1
Total	68	54	31	23

**Figure 2:**
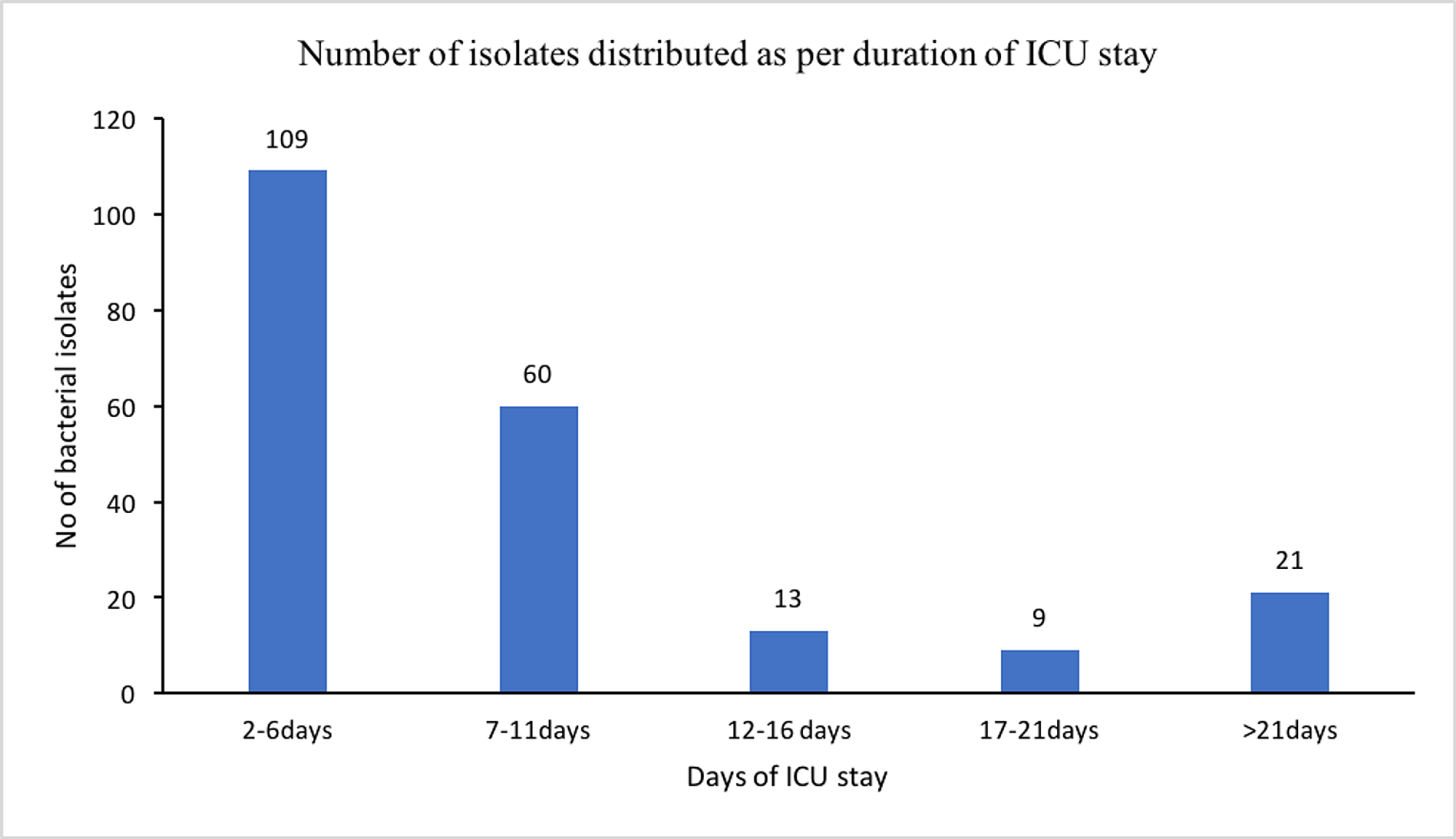
Total number of bacterial isolates as per the duration of stay in ICU

Two hundred nine bacteria were isolated from 121 cases (a rectal swab of one patient did not yield any isolate) with *E. coli* the most common isolate. When the study subjects were distributed according to days of hospitalization, it was seen that the number of patients dropped as the days of hospitalization increased. Subsequently, most bacterial isolates were obtained during two to six days of stay in the ICU (Figure 2). However, the number of bacterial genera isolated per patient increased with the days of stay in the ICU (Table 3).

**Table 3:** Showing the number of bacteria genera isolated per patient based on days of ICU stay *rectal swab of one patient did not yield any isolate

No of different genera isolated	2-6days (%)	7-11days (%)	12-16days (%)	17-21days (%)	>21days (%)	Total
1	30 (63.9)	9 (19.1)	4 (8.5)	1 (2.1)	3 (6.4)	47
2	32 (50.8)	19 (30.2)	3 (4.8)	4 (6.4)	5 (7.9)	63
3	2 (22.2)	3 (33.3)	1 (11.1)	0	3 (33.3)	9
4	0	1(50)	1(50)	0	0	2
Total	64(52.9)	32(26.4)	9 (7.4)	5(4.1)	11(9.1)	121*

Carbapenem-resistant organisms (CRO)

**Figure 3:**
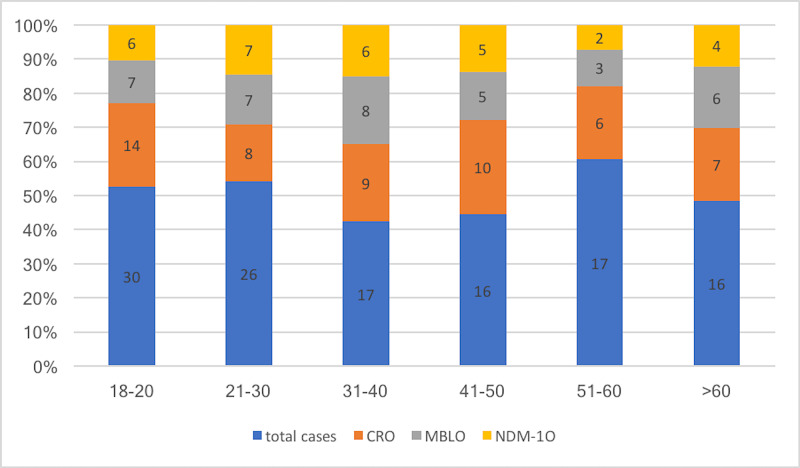
Showing age distribution of patients with different isolates CRO = carbapenem-resistant organisms, MBLO = metallo-β-lactamase producing organisms, NDM = New Delhi metallo-ß-lactamase

Seventy-nine of 209 (37.8%) isolates were found to be carbapenem-resistant (Table 4). A total of 54/122 (44.3%) patients harbored carbapenem-resistant organisms (Figure 3). Among the patients harboring CRO, the mean duration of stay in the ICU exclusively and the total days of stay in the hospital were respectively 8.4 and 11.5 days, respectively. The same values for those not harboring CRO were 7.9 and 10.3 days. 

**Table 4:** Types of isolates CRO = carbapenem-resistant organisms, MBL = metallo-β-lactamase

Bacterial isolate	No. of isolate (% of total)	CRO (% of the bacterial species isolated)	MBL producer (% of CRO)	NDM-1 carrying (% of MBL producers)
Escherichia coli	185 (88.5)	64 (34.6)	41 (64.1)	30 (73.2)
Klebsiella pneumoniae	17 (8.1)	12 (70.6)	9 (75)	7 (77.8)
Pseudomonas aeruginosa	5 (2.4)	3 (60)	3 (100)	3 (100)
Proteus mirabilis	2 (0.95)	0	0	0
Total	209	79 (37.8)	53 (67.1)	40 (75.5)

The maximum number of CRO was found in the 41-50 years age group (62.5%) followed by 31-40 years (52.9%). Other age groups like 18-20 years, 21-30 years, 51-60 years, and >60 years had 46.7%, 44.4%, 35.3%, and 43.7% CRO respectively. However, no age group showed a significant association (p >0.05). There was no predilection for either gender (p >0.05) (Table 2).

**Figure 4:**
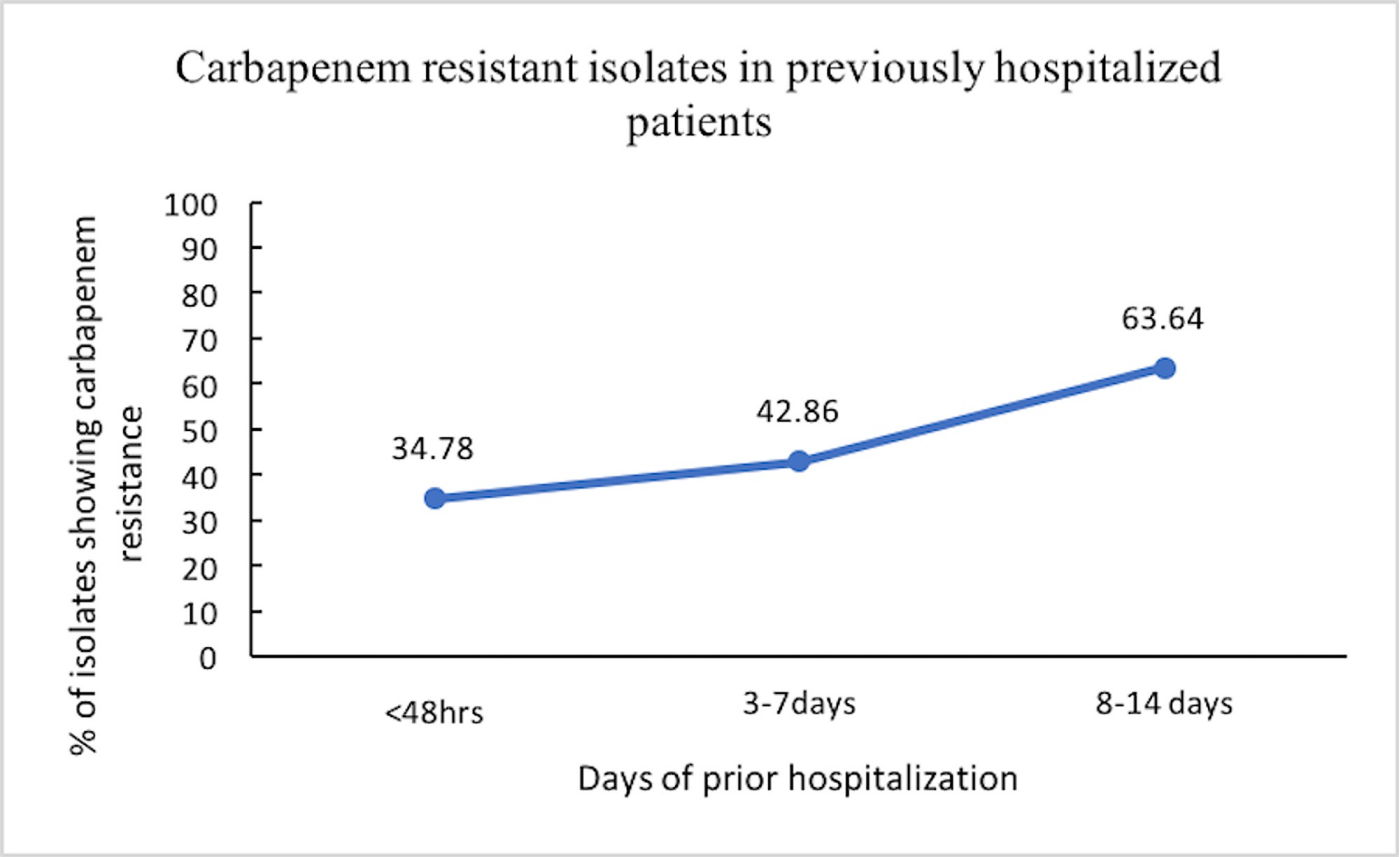
Showing patients with a history of prior hospitalization harbouring carbapenem-resistant organisms (CRO) (%)

There was a significant increase in the proportion of patients harboring CRO from days two to six (38.5%) to days seven to 11 (62.5%) of ICU stay (p <0.05). The rest was not significant. An increase in the duration of hospitalization just before being admitted to the ICU was associated with an increase in the prevalence of CRO in the gut flora (Figure 4).

Metallo-β-lactamase (MBL) producers 

Out of 79 carbapenem-resistant organisms, 53 isolates (67.1%) were found to be metallo-β-lactamase producers (Table 4). A total of 36 patients harbored metallo-β-lactamase producers (Figure 4). The mean duration of stay in the ICU and hospital of these patients harboring MBL producers was 10.8 and 14.6 days, respectively. Both these values were higher than that of those not harboring MBL producers (7.8 and 10.5 days, respectively). 

**Figure 5:**
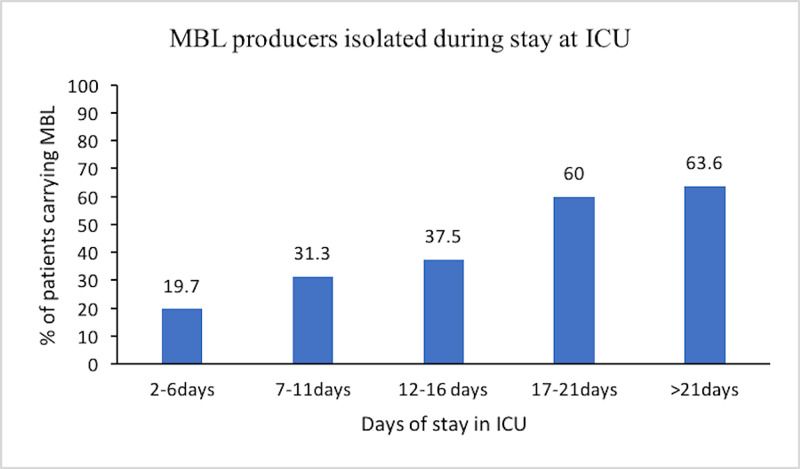
Showing percentage of patients harboring metallo-β-lactamase (MBL) producers

The number of patients harboring MBL producers was highest in the age group 31-40 years (47.1%), followed by >60 years (37.5%), and the least number of patients harboring MBL producers were in the age group 51-60 years (17.6%). Other age groups like 18-20 years, 21-30 years, and 41-50 years had 23.3%, 26.9%, and 31.3% of patients with MBL-producing bacteria. The percentage of patients harboring MBL producers increased with the increase in the duration of stay in ICU (p >0.05) (Figure 5).

New Delhi metallo-β-lactamase 1 (NDM-1)

A total of 40 (75.5%) gut colonizers were carrying the NDM-1 gene (Table 4). A total of 30/122 (24.6%) patients admitted to ICU harbored bacteria carrying the NDM-1 gene. It was most frequently detected in the age group 31-40 years 6/17 (35. 3%) and was least common in the age group 51-60 years 2/17 (11.8%) (Figure 3).

Different morphotypes of *E. coli* were correlated with the production of metallo-β-lactamase. Out of 185, 120 (64.9%) were flat, 56 (30.3%) were mucoid, six (3.2%) were non-lactose-fermenting and three (1.6%) were small colony variants. All small colony variants (100%) and 66.7% of non-lactose fermenting (NLF) variants were MBL producers and carried the NDM-1 gene. Only 21/120 (17.5%) flat colony were MBL producers with 11/120 (9.2%) carrying the NDM-1 gene, and 13/56 (23.2%) mucoid morphotype were MBL producers with 12/56 (21.4 %) carrying NDM-1 gene (Table 5). 

**Table 5:** Showing distribution of carbapenem resistance, MBL production, and NDM-1 gene in Escherichia coli morphotypes MBL = metallo-β-lactamase, NDM-1 = New Delhi metallo-ß-lactamase 1, NLF = non-lactose fermenting

Morphotypes of Escherichia coli (%)	Total isolates (%)	Carbapenem resistant isolates (%)	P-value	MBL producers (%)	P-value	Presence of NDM-1 gene (%)	P-value
Flat	120 (64.9)	37 (30.8)	0.7295 0.0285^*^ 1.000	21 (56.8)	0.5643 1.000 1.000	11 (52.4)	0.0238^*^ 1.000 1.000
Mucoid	56 (30.3)	19 (33.9)		13 (68.4)		12 (92.3)	
NLF	6 (3.2)	5 (83.3)		4 (80.0)		4 (100)	
Small cell	3 (1.6)	3 (100)		3 (100)		3 (100)	
Total	185	66 (35.7)		41 (62.1)		30 (73.21)	

All the metallo-β-lactamases were simultaneously resistant to amikacin, ciprofloxacin, third-generation cephalosporins, and amoxicillin + clavulanic acid.

Risk factors for carriage of carbapenem-resistant organisms (CRO)

Use of invasive devices, consumption of ≥3 groups of antibiotics, hospitalization during the previous six months, comorbidities, and hospital stay for ≥48 hours before ICU admission is significantly associated with colonization with CRO. 

Of the 122 patients admitted in the ICU, 67 (54.9%) expired. Of the expired patients, 36 (53.7%) harbored CRO, 17 (25.4%) carried MBL producing colonizers, seven (10.4%) harbored bacteria carrying the NDM-1 gene.

## Discussion

During the study period, 122 adult patients consisting of 54 females and 68 males admitted to the ICU for ≥ 48 hrs were recruited for surveillance of the NDM-1 gene in gut colonizers.

The majority of the patients included in the study were below 30 years of age. There was a high rate of ICU admission of patients with extra-pulmonary tuberculosis, obstetric complications, road traffic accidents, head injuries, blunt trauma abdomen, tetanus, and Guillain-Barre syndrome. Fractures, cerebrovascular accidents, surgery for various cancers, type 2 diabetes mellitus and its complications were the major causes of ICU admission of patients above 40.

This study found a very high proportion of carbapenem-resistant organisms (CRO), making 44.3% of the total isolates. Various studies from India reported a prevalence of CRO in the range of 1.8% to 51% [9]. One study by Kumar et al. reported up to 73% [10]. Our study is in accordance with these findings. However, some studies reported a low level of CRO, even up to 0.5% [3,11,12]. The markedly high degree of resistance of gut colonizers to carbapenem in this study can be attributed to geographical variation, different profiles of antibiotic consumption, and the difference in the predominance of bacteria isolated. It may also be because the study was carried out in patients admitted in the ICU, where both antibiotic consumption and development of resistance is highest among all healthcare settings. 

The most common isolate in this study was *E. coli*, but carbapenemase production was maximally seen in *Klebsiella pneumoniae* (70.6%), followed by *Pseudomonas aeruginosa* (60%) and *E. coli* (34.6%). Our study, like other similar studies, reported *K. pneumoniae* the commonest carbapenemase-producing organism [5,9,13-15]. However, some studies reported organisms other than *K. pneumoniae* as a common carbapenemase producer [3,10,16,17].

The prevalence of both CRO and MBL producing organisms increased with an increase in the duration of ICU/hospital stay. This finding was further emphasized with an epidemiological study of bacterial colonizers in the intensive care unit, where this was shown to be statistically significant [18].

As previously mentioned, prior hospitalization was a risk factor for the development of carbapenem resistance. This increased risk was demonstrated by a steep increase in the slope of the curve, denoting resistance patterns with days of prior hospitalization (Figure 4). Since it had been seen that in critically ill patients, colonization of the respiratory and gastrointestinal tract with nosocomial flora occurs within 48-72 hrs after admission, to be variably followed by invasive disease, it may be concluded that beyond seven days of hospital stay, maximum patients developed colonizers and also subsequent clinical symptoms. 

Forty of 53 (75.5%) of the MBL-producing isolates included in the study carried the NDM-1 gene. So it could be concluded that NDM-1 would be the most common mecha­nism of carbapenem resistance. This is also indicated in a study by Bryan [19].

Among *E. coli*, four morphotypes were identified, which were a flat type of lactose fermenting colonies, a mucoid type of lactose fermenting colonies, non-lactose fermenting colonies, and the small colony variants. This was done taking into account that different morphotypes exhibit different degrees of resistance to the same antibiotic as described in studies by Malone et al. [20].

Many studies have shown that the use of invasive devices, three or more classes of antibiotics, hospitalization during the previous six months, comorbidities, and hospital stay for ≥48 h were significantly associated with the development of CRO carriage [5,9,11,12]. While the use of multiple antibiotics causes selection pressure, invasive devices, and comorbid conditions lead to prolonged hospitalization resulting in the gastrointestinal carriage of CRO [11,12]. Though some studies reported the age of patients as being a risk factor, we did not find this result [5].

Patients colonized with CRO showed increased mortality than patients not colonized with CRO (53.7%, p = 0.027). Several studies corroborate our study, showing a mortality rate ranging from 30%-75% [5,21].

## Conclusions

With the channel of time growth, co-resistance of different extended-spectrum blaNDM-1 genes is annoying as the co-existence of multiple genes hinders the detection of MBL-producers, and complicates the treatment strategy for clinicians. Moreover, a high plasmid burden was found. These plasmids are involved in gene transfer, and they also carry additional antibiotic resistance genes, including New Delhi metallo-ß-lactamase. This study confirmed that patients with gastrointestinal carriage of CRO had a risk for infections with multidrug-resistant organisms, prolonged hospital stays, and more adverse outcomes. It would be wise to identify these carrier patients, especially in ICU settings, and isolate them under infection prevention strategy. This step would be again helpful to inhibit the spread of such organisms among different patients and finally in society.
